# Epidermal Inclusion Cyst Presenting as a Palpable Scrotal Mass

**DOI:** 10.1155/2012/498324

**Published:** 2012-10-09

**Authors:** Andres F. Correa, Bishoy A. Gayed, Mitchell E. Tublin, Anil V. Parwani, Jeffrey R. Gingrich

**Affiliations:** ^1^Department of Urology, University of Pittsburgh, 3471 5th Ave, Suite 700 Kaufmann Bldg, Pittsburgh, PA 15213, USA; ^2^Department of Radiology, University of Pittsburgh, 3950 Lothrop St, Presbyterian Hospital South Tower 200, Pittsburgh, PA 15213, USA; ^3^Department of Pathology, University of Pittsburgh, 230 Centre Ave, Room WG 02.10 Shadyside Hospital, Pittsburgh, PA 15213, USA

## Abstract

We report a scrotal epidermal inclusion cyst located outside the median raphe which a rare entity in the absence of trauma and few cases have been reported. 47 year old male presents with a complaint of right sided testicular swelling and discomfort. On examination a 3 cm mass was palpated between the scrotum and the medial thigh on the subcutaneous tissue with a positive slip sign. Complete surgical excision of the cyst was performed. Histopathology confirmed epidermal inclusion cyst with no evidence of malignancy.

## 1. Introduction

Epidermoid inclusion cysts (EIC) are rare, benign cysts which occur from implantation of epidermal tissue into the dermis or subcutaneous tissues or in the testicle [1]. This abnormal localization can occur from developmental closure of the median raphe to include the epidermal tissue or through traumatic implantation [1]. These EIC are usually located along the midline and have been reported from the distal penis to the anus [1]. The presentation in the scrotum is usually an asymptomatic, firm, freely moveable extra-testicular mass developing between the second and forth decade of life [2]. We report a case of an extra-testicular epidermoid inclusion cyst with an atypical presentation away from the midline as well as a review of the diagnosis, evaluation and management of these unique scrotal anomalies.

## 2. Case Report

A healthy, 47-year-old male presented with testicular discomfort and a palpable nodule in the upper scrotum just medial to the thigh. He reported that the nodule had been present for nearly seven years and had been slowly enlarging over the last few years. The mass had been initially asymptomatic, until recently when he began to experience some discomfort during exercise along with other activities. He denied any history of trauma or any prior surgeries to the area.

**Figure 1:**
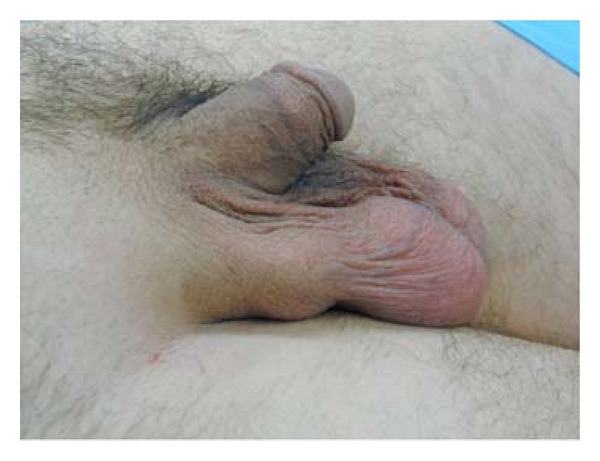
Physical appearance of right extra-testicular mass on 47 year old male.

**Figure 2:**
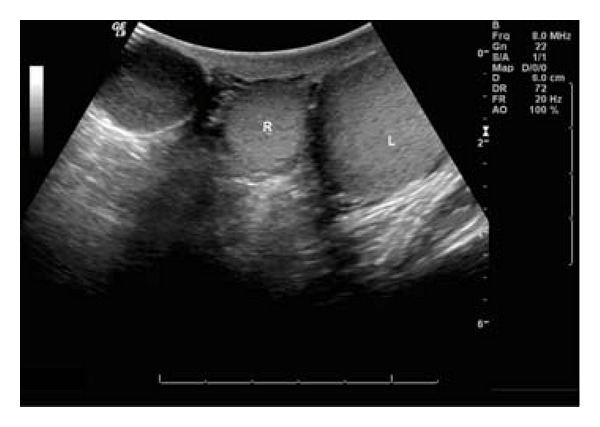
Coronal oblique sonogram shows an oval solid hypoechoic mass (arrow) separate from the right and left testes (T).

On physical exam, a palpable 3 cm soft structure was present in the right scrotum as shown in Figure 1. It was minimally tender and mobile within the subcutaneous tissue, not adherent to the skin or spermatic cord. A scrotal ultrasound revealed a uniform, hypoechoic right extra-testicular lesion. The lesion appeared to be avascular. The mass did not originate from the scrotal wall, but was abutted by the lateral aspect of the right testis as shown in Figure 2.

**Figure 3:**
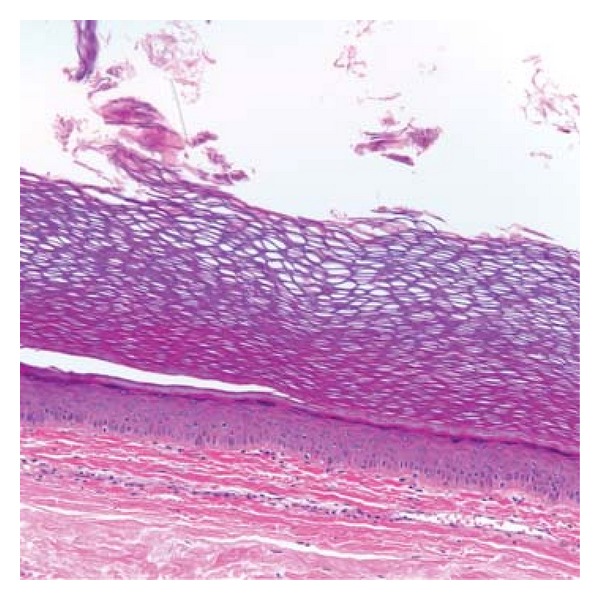
High power view of epidermal inclusion cyst wall demonstrating benign squamous epithelium with keratin formation (200x).

 Given the low malignant potential, we did not send testicular markers and proceeded with excision of the mass. The cyst was found to be superior and lateral to the right testicle, but not originating from it. It was excised without difficulty. Intraoperative findings revealed the mass to be covered with a thin sac that contained necrotic, proteinaceous material. Pathological findings revealed benign epidermal inclusion cyst as shown in Figure 3. The patient was seen in 1 month followup and recovered with no postoperative complications.

## 3. Discussion

 EIC are benign tumors consisting of a sac lined by stratified squamous epithelium filled with laminated keratin, cholesterol crystals, and debris [2]. The cysts are usually asymptomatic unless they become large enough to interfere with function, become infected, or rupture causing inflammation of adjacent structures [2]. Usually these masses are midline and most reports place them along the median raphe. In our patient, the mass presented as a palpable nodule in between the scrotum and medial thigh. Our patient denied any history of scrotal trauma to explain the aberrant location.

 Ultrasound has become the primary imaging modality of intrascrotal lesions. On ultrasound, epidermoid inclusion cysts appear as well-circumscribed round or oval hypoechoic lesions with scattered echogenic reflectors and no evidence of internal blood flow on doppler. Lee et al. proposed five characteristic sonographic patterns of EIC after reviewing 24 patients with EIC in a variety of anatomical locations [3]. They were described as ovoid, lobulated or tubular in shape and classified as (I) alternating hypoechoic and hyperechoic eccentric rings (II) target sign (hyoechoic lesion with hyperechoic center (III) hypoechoic lesion with scattered echogenic reflectors (IV) inhomogeneous lesion, and V. areas of varying echogenicity. In their series, seventy-one percent were ovoid, 21% lobulated, and 8% tubular. The classification showed Type I—13%, II—8%, III—42%, IV—29% and V—8%. Nine patients presented with histologic evidence of rupture, frequently showing lobulated shape or internal flow on color Doppler [3]. 

 In addition to ultrasound, magnetic resonance imaging (MRI) has become a popular tool in the evaluation of scrotal masses. On MRI, EICs are described as high intensity well defined solid masses surrounded by a low signal capsule on T2-weighted imaging [4]. With the administration of gadolinium there is a lack of enhancement on T1 imaging consistent with the avascular nature of the mass [4]. 

 Although, scrotal extra-testicular lesions are an uncommon occurrence and benign in nature, the clinician should be cautious when it comes to the evaluation and management of these masses. Malignant extra-testicular lesions are uncommon but have been reported and include lymphoma, liposarcoma, fibrosarcoma or metastatic disease. The clinical debate has always been how to differentiate benign from malignant lesions. Even with the advancements of imaging modalities and characteristic features of epidermoid cysts ultrasound and MRI cannot entirely exclude the possibility of malignancy. 

## 4. Conclusion

 We share a case of an epidermoid inclusion cyst of the testicle found in an atypical location. The mass presented with benign features on physical exam and on ultrasound imaging. Because of the increasing discomfort along with the atypical location, we elected to proceed with excision of the mass. Epidermoid inclusion cysts of the testicle are very rare, benign masses, with low malignant potential. When physical and sonographic features are suggestive of epidermoid inclusion cyst, the management should be scrotal exploration with testicular sparing resection as to prevent infection or rupture complications and to avoid missing a possible malignancy.
